# Risk Factors for Acute Kidney Injury and In-Hospital Mortality in Patients Receiving Extracorporeal Membrane Oxygenation

**DOI:** 10.1371/journal.pone.0140674

**Published:** 2015-10-15

**Authors:** Sung Woo Lee, Mi-yeon Yu, Hajeong Lee, Shin Young Ahn, Sejoong Kim, Ho Jun Chin, Ki Young Na

**Affiliations:** 1 Division of nephrology, Department of internal medicine, Seoul National University Bundang Hospital, Seongnam, Gyeonggi-do, Korea; 2 Division of nephrology, Department of internal medicine, Seoul National University Hospital, Seoul, Korea; 3 Department of Internal Medicine, Seoul National University College of Medicine, Seoul, Korea; University of Sao Paulo Medical School, BRAZIL

## Abstract

**Background and Objectives:**

Although acute kidney injury (AKI) is the most frequent complication in patients receiving extracorporeal membrane oxygenation (ECMO), few studies have been conducted on the risk factors of AKI. We performed this study to identify the risk factors of AKI associated with in-hospital mortality.

**Methods:**

Data from 322 adult patients receiving ECMO were analyzed. AKI and its stages were defined according to Kidney Disease Improving Global Outcomes (KDIGO) classifications. Variables within 24 h before ECMO insertion were collected and analyzed for the associations with AKI and in-hospital mortality.

**Results:**

Stage 3 AKI was associated with in-hospital mortality, with a hazard ratio (HR) (95% CI) of 2.690 (1.472–4.915) compared to non-AKI (p = 0.001). The simplified acute physiology score 2 (SAPS2) and serum sodium level were also associated with in-hospital mortality, with HRs of 1.02 (1.004–1.035) per 1 score increase (p = 0.01) and 1.042 (1.014–1.070) per 1 mmol/L increase (p = 0.003). The initial pump speed of ECMO was significantly related to in-hospital mortality with a HR of 1.333 (1.020–1.742) per 1,000 rpm increase (p = 0.04). The pump speed was also associated with AKI (p = 0.02) and stage 3 AKI (p = 0.03) with ORs (95% CI) of 2.018 (1.129–3.609) and 1.576 (1.058–2.348), respectively. We also found that the red cell distribution width (RDW) above 14.1% was significantly related to stage 3 AKI.

**Conclusion:**

The initial pump speed of ECMO was a significant risk factor of in-hospital mortality and AKI in patients receiving ECMO. The RDW was a risk factor of stage 3 AKI.

## Introduction

Although extracorporeal membrane oxygenation (ECMO) has been used in severe cardiopulmonary diseases since the 1970s, the outcome in the early ECMO era was not satisfactory [1–3]. Since this time, major advances in critical care [4] and technical aspects [5] have been made. The 2009 H1N1 influenza pandemic gave birth to several studies that suggested improved outcomes of modern ECMO [6–8]. In the Korean epidemic of Middle East respiratory syndrome (MERS), ECMO played a key role in treating critically ill patients.

ECMO enables an efficient oxygenation and elimination of carbon dioxide. ECMO can be operated in two different modes: a venovenous (VV) mode for ventilatory failure and a veno-arterial (VA) mode for respiratory and cardiac support, however, a number of complications could impinge upon the potential benefit of ECMO, and acute kidney injury (AKI) is the most frequently reported problem [9, 10].

Three major sets of criteria have been proposed to define AKI RIFLE (the Risk of renal failure, Injury to the kidney, Failure of kidney function, Loss of kidney function and End stage kidney disease), the Acute Kidney Injury Network (AKIN), and the Kidney Disease Improving Global Outcomes (KDIGO) criteria. Lin et al. retrospectively analyzed 46 patients who were treated with ECMO and demonstrated the good discriminatory power of the RIFILE criteria for in-hospital mortality [area under the receiver operator curve (AUROC), 0.868, p < 0.001] [11]. Yan et al. analyzed 67 patients receiving ECMO support and showed similar results; the reported AUROC for in-hospital mortality was 0.738 (p = 0.001) and 0.799 (p <0.001) for RIFLE and AKIN criteria, respectively [12]. However, all of the previous studies have mainly focused on the association between AKI and mortality, and none of them have evaluated the potential risk factors of AKI in adult patients [11–14]. Therefore, we performed this retrospective cohort study to explore the risk factors for AKI and in-hospital mortality in patients receiving ECMO support.

## Materials and Methods

### Study population

We performed a retrospective cohort study in adult patients who were 15 years or older and received ECMO support at Seoul National University Bundang Hospital and Seoul National University Hospital, which are the two tertiary care hospitals. The study protocol complied with the Declaration of Helsinki and received full approval from the institutional review boards at both Seoul National University Bundang Hospital (B-1412/278-112) and Seoul National University Hospital (J-1503-003-651). Informed consent was waived because patient records/information were anonymized and de-identified prior to analysis. No extramural funding was used to support this work. A total of 681 patients consecutively received ECMO support from January 2005 to November 2014. Patients were excluded from the analysis if they were under 15 years of age (n = 109), if they received ECMO support for less than 24 h (n = 90), if they received ECMO insertion from other hospitals (n = 4), if they had end-stage renal disease or their initial serum creatinine levels were above 353.6 μmol/L (n = 13), or if they had been receiving continuous renal replacement therapy when ECMO were initiated (n = 66), if they initiated continuous renal replacement therapy on the date of ECMO insertion (n = 77). Therefore, 322 patients were ultimately analyzed in the present study.

### Measurements and Definitions

The physiologic and laboratory data within 24 h before ECMO initiation were collected retrospectively through a review of the electronic medical records. The clinical parameters that were recorded included the following: age, sex, causes of admission; causes of ECMO support, mode of ECMO, whether to perform cardiopulmonary resuscitation within 24 h, use of an intra-aortic balloon pump (IABP), ECMO settings, duration of ECMO, urine output, and ventilator settings. Initial blood findings, including blood urea nitrogen (BUN), total bilirubin, albumin, white blood cells, hemoglobin level, platelet number, red cell distribution width (RDW), sodium, potassium, chloride, and C-reactive protein (CRP) were measured. For the severity index, we used the Simplified Acute Physiology Score 2 (SAPS2) [15]. To calculate the SAPS2, the worst values during the first 24 h before ECMO initiation were used.

AKI and the stage of its severity were defined according to the guidelines proposed by KDIGO [16]. AKI was defined in a case with either an increase in serum creatinine by ≥ 26.5 μmol/L or ≥ 1.5 times the baseline within 48 h. The changes in serum creatinine according to the AKI stages were as follows: stage 1, an increase of more than or equal to 26.5 μmol/L or an increase to more than or equal to 1.5- to 2-fold of the baseline; stage 2, an increase to more than 2- to 3-fold of the baseline; stage 3, an increase to more than 3-fold of the baseline or more than or equal to 353.6 μmol/L with an acute increase of at least 44.2 μmol/L or on renal replacement therapy. The maximum AKI stage reached during ECMO support was used to define the incidence of AKI [17]. In-hospital mortality was determined whether a death certificate had been issued or not at 90 d after ECMO insertion. The applied ECMO console was composed of a centrifugal pump and membrane oxygenator. The products utilized included CAPIOX EBS (Terumo Corporation, Tokyo, Japan) and QUADROX PLS (Maquet, Hirrlingen, Germany).

### Statistical analysis

The values were expressed as the mean ± standard deviation in continuous variables and n (%) in categorical variables. For the severely skewed variables, such as follow-up duration, the median (interquartile range, IQR) was used. The difference was analyzed by an independent t-test in continuous variables and chi-square test in categorical variables. For the estimated survival, the Kaplan-Meier method was employed, and the statistical significance was calculated using the log-rank test. For multivariate analysis, logistic regression analysis for AKI and Cox-proportional hazard analysis for in-hospital mortality were carried out. The variables in the multivariate analysis were chosen by p <0.05 in the univariate analysis. Calibration was done using the Hosmer-Lemeshow goodness-of-fit test to compare the numbers of predicted and observed in-hospital mortality and AKI. Discrimination was analyzed using AUROC. The best threshold was calculated by obtaining the best Youden index (sensitivity + specificity—1). We consider p <0.05 to be statistically significant. All of the analyses were performed using the SPSS statistics software (version 22, IBM, USA).

## Results

The mean age of the study participants was 60.3 ± 15.3 years and 213 (66.1%) of the participants were male. The reasons for admission were cardiovascular disease (203, 63.0%), lung disease (49, 15.2%), malignancy (35, 10.9%) and others (35, 10.9%). One hundred and thirty seven (42.5%) patients had received cardiopulmonary resuscitation within 24 h prior to ECMO initiation. After the median (IQR) 2 (0–10) days of admission, the patients received ECMO insertion because of cardiotomy (31, 9.6%), non-operative cardiovascular causes (185, 57.5%), adult respiratory distress syndrome (ARDS) (43, 13.4%), non-ARDS lung causes (44, 13.7%) and other causes (19, 5.9%). Two hundred and thirty (71.4%) and 92 (28.6%) patients received VA and VV ECMO support, respectively. One hundred and six (32.9%) patients were undergoing IABP on the date of ECMO insertion. The median (IQR) duration from ECMO initiation to death or discharge was 21 (8–40) days. The incidence of AKI comprising all KDIGO grades was 82.3%. In-hospital mortality was 51.6%. The median (IQR) durations for AKI and in-hospital mortality were 2 (1–7) days and 9 (4–23) days, respectively.

**Fig 1:**
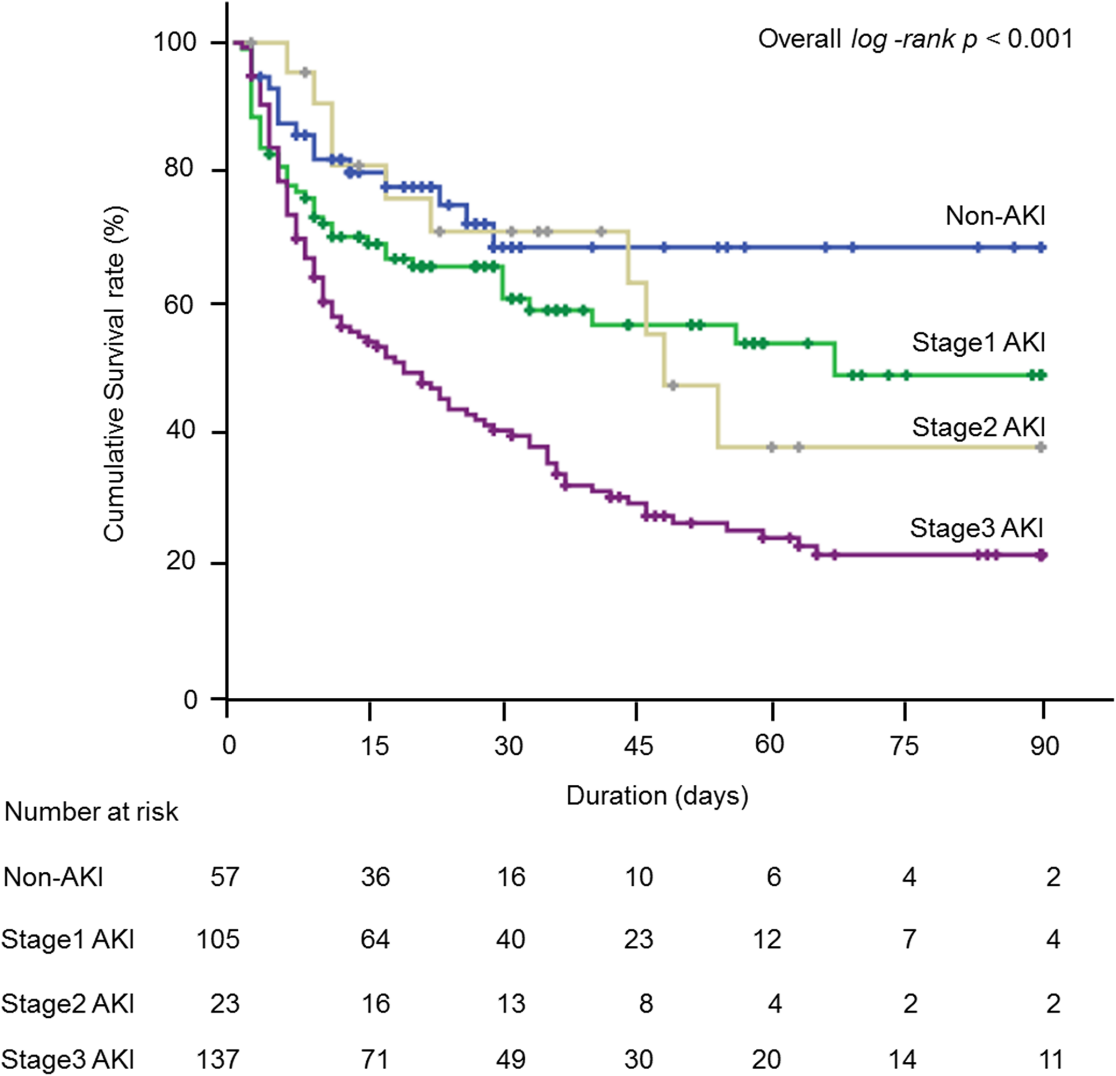
Kaplan-Meier survival curves for in-hospital mortality according to the stages of acute kidney injury. AKI, acute kidney injury.

We explored the factors associated with in-hospital mortality. AKI developed less frequently in the survivor group than in the non-survivor group. Moreover, stage 3 AKI developed significantly less in the survivors than in the non-survivors. SAPS2 and the serum sodium level were significantly lower in the survivors than in the non-survivors. Ventilator settings, such as positive end expiratory pressure and peak inspiratory pressure before ECMO insertion, did not affect the survival rate. The ECMO pump speed was significantly lower in the survivors than in the non-survivors. Age, causes of admission, causes of ECMO support, mode of ECMO, use of IABP, length of stay before ECMO insertion, duration of ECMO support, initial urine output, BUN, creatinine, RDW and CRP were associated with in-hospital mortality (Table 1). We performed a multivariate Cox-proportional hazard regression analysis to adjust confounding effects among the selected variables. Compared to the non-AKI group, the stage 3 AKI group significantly increased the risk of in-hospital mortality whereas the stage 1 and 2 AKI groups did not (Table 2). In the Kaplan-Meier survival curves according to the stages of AKI, the estimated mean (95% CI) survival in the non-AKI group and the stage 1, 2, and 3 groups were 65.7 (55.2–76.2) days, 54.0 (45.8–62.3) days, 53.8 (38.7–69.0) days and 33.6 (27.9–39.4) days, respectively (p < 0.001 by log-rank test). In the post-hoc analysis, the stage 3 AKI group, but not the stage 1 (p = 0.14) or 2 (p = 0.43) AKI groups, showed a significant difference in survival compared with the non-AKI group (Fig 1). With every increment in SAPS2, serum sodium level, and ECMO pump speed (1 score in SAPS2, 1 mmol/L in serum sodium level, and 1,000 rpm in ECMO pump speed), the risks of in-hospital mortality were increased, with HRs (95% CI, p-value) of 1.02 (1.004–1.035, 0.01), 1.042 (1.014–1.070, 0.003) and 1.333 (1.020–1.742, 0.04), respectively (Table 2). We performed a calibration and discrimination analysis of SAPS2, serum sodium level, and ECMO pump speed to predict in-hospital mortality. All three variables were well-calibrated. The AUROC analysis showed the discriminative power of these variables. The cut-off values of SAPS2, serum sodium level, and ECMO pump speed for in-hospital mortality were a score of 69.5, 147.6 mmol/L, and 2.19 x 10^3^ rpm, respectively (Table 3).

**Table 1:** Patient characteristics according to survival status.

		Survivor (n = 156)	Non-survivor (n = 166)	*p*
Age (years)		58.3 ± 15	62.3 ± 15.4	0.02
Male sex		95/156 (60.9)	118/166 (71.1)	0.054
Center 2		82/156 (52.6)	92/166 (55.4)	0.61
Causes of admission				0.002
	Cardiovascular disease	109/156 (69.9)	94/166 (56.6)	0.01
	Lung disease	21/156 (13.5)	28/166 (16.9)	0.40
	Malignancy	7/156 (4.5)	28/166 (16.9)	<0.001
	Others	19/156 (12.2)	16/166 (9.6)	0.46
Causes of ECMO support				0.02
	ARDS	12/156 (7.7)	31/166 (18.7)	0.004
	Non-ARDS lung causes	22/156 (14.1)	22/166 (13.3)	0.82
	Post-cardiotomy	14/156 (9.0)	17/166 (10.2)	0.70
	Non-operative cardiac causes	101/156 (64.7)	84/166 (50.6)	0.01
	Others	7/156 (4.5)	12/166 (7.2)	0.30
ECMO VA mode		121/156 (77.6)	109/166 (65.7)	0.02
CPR within 24 hours		61/156 (39.1)	76/166 (45.8)	0.23
IABP use		62/156 (39.7)	44/166 (26.5)	0.01
Length of stay before ECMO insertion (days)		6.5 ± 12.6	10.7 ± 21.8	0.03
Initial ECMO settings				
	Pump speed (10^3^ rpm) ^a^	2.2 ± 0.6	2.4 ± 0.7	0.005
	Blood flow rate (L/min)	3.1 ± 0.8	3.1 ± 1	0.88
	Blood flow rate in VA (L/min)	3.1 ± 0.8	3.2 ± 1.1	0.48
	Blood flow rate in VV (L/min)	3.2 ± 0.8	2.9 ± 0.8	0.11
ECMO duration (days)		6.4 ± 7.6	10.8 ± 10.7	<0.001
Initial urine output (L/day)		4.7 ± 3.8	3.9 ± 2.8	0.03
Initial ventilator settings				
	PEEP (cmH_2_O) ^a^	5.6 ± 2.7	6 ± 2.3	0.17
	PIP (cmH_2_O) ^a^	16.9 ± 5.7	17.6 ± 6.1	0.33
Initial laboratory findings				
	Blood urea nitrogen (mmol/L)	16.7 ± 9.4	20.6 ± 13.2	0.002
	Creatinine (μmol/L)	105.5 ± 47	118.2 ± 54.1	0.03
	Total bilirubin (μmol/L) ^a^	28.7 ± 27.7	31.6 ± 31.6	0.40
	Albumin (g/L) ^a^	28.1 ± 6.3	26.7 ± 6.3	0.053
	White Blood Cells (x10^3^/μL)	13.5 ± 6.6	14.1 ± 8.5	0.55
	Hemoglobin (g/dL)	11.2 ± 2.4	10.8 ± 2.3	0.13
	Platelet (x10^3^/μL)	137.7 ± 71.1	150.8 ± 94.1	0.16
	RDW (%)	14.3 ± 1.4	14.8 ± 2	0.01
	Sodium (mmol/L)	140.2 ± 6.7	142.6 ± 7.4	0.004
	Potassium (mmol/L)	3.9 ± 0.7	4 ± 0.8	0.17
	Chloride (mmol/L)	105.8 ± 7.4	106.1 ± 7.2	0.67
	C-reactive protein (nmol/L) ^a^	57 ± 73.1	78.3 ± 85	0.02
Initial SAPS2 ^a^		58.1 ± 14.3	63.9 ± 15.2	0.001
AKI		114/156 (70.1)	151/166 (91.0)	<0.001
	Non- AKI	42/156 (26.9)	15/166 (9.0)	<0.001
	Stage 1	63/156 (40.4)	42/166 (25.3)	0.004
	Stage 2	13/156 (8.3)	10/166 (6.0)	0.421
	Stage 3	38/156 (24.4)	99/166 (59.6)	<0.001

Values are expressed as mean ± standard deviation in continuous variables and n/total (%) in categorical variables. Difference was analyzed by t-test in continuous variables and chi-square test in categorical variables. ECMO, extracorporeal membrane oxygenation; ARDS, acute respiratory distress syndrome; VA, venoarterial; CPR, cardiopulmonary resuscitation; IABP, intraarterial balloon pump; PEEP, positive end expiratory pressure; PIP, peak inspiratory pressure; RDW, red cell distribution width; SAPS2, Simplified acute physiology score 2; AKI, acute kidney injury.

^a^The total numbers of survivor/non-survivor of pump speed, PEEP, PIP, total bilirubin, albumin, C-reactive protein and initial SAPS2 were 156/165, 146/161, 146/160, 156/163, 156/164, 139/151 and 151/161, respectively.

**Table 2:** Multivariate Cox-proportional hazard regression analysis for in-hospital mortality.

		Hazard ratio (95% CI)	*p*
Age (every 1 year increase)		0.997 (0.983–1.011)	0.66
Sex (Male vs. Female)		1.149 (0.775–1.705)	0.49
Causes of admission (vs. Cardiovascular disease)			0.20
	Lung disease	1.389 (0.574–3.362)	0.47
	Malignancy	1.432 (0.728–2.819)	0.30
	Others	0.633 (0.287–1.396)	0.26
Causes of ECMO support (vs. ARDS)			0.21
	Non-ARDS lung cause	0.707 (0.320–1.562)	0.39
	Post-cardiotomy	1.652 (0.572–4.768)	0.35
	Non-operative cardiac cause	0.933 (0.357–2.435)	0.89
	Others	1.649 (0.665–4.084)	0.28
ECMO mode (VA vs. VV)		1.130 (0.498–2.568)	0.77
IABP use (yes vs. no)		0.771 (0.501–1.186)	0.24
Length of stay before ECMO insertion (every 1 day increase)		1.003 (0.993–1.013)	0.55
ECMO pump speed (every 10^3^ rpm increase)		1.333 (1.020–1.742)	0.04
ECMO duration (every 1 day increase)		0.994 (0.974–1.014)	0.53
Initial urine output (every 1L/day increase)		0.939 (0.879–1.003)	0.06
Initial laboratory findings			
	Blood urea nitrogen (every 1 mmol/L increase)	0.985 (0.966–1.004)	0.12
	Creatinine (every 1 μmol/L increase)	1.003 (0.998–1.008)	0.20
	RDW (every 1% increase)	0.970 (0.868–1.085)	0.60
	Sodium (every 1 mmol/L increase)	1.042 (1.014–1.070)	0.003
	C-reactive protein (every 1 nmol/L increase)	1.000 (0.997–1.002)	0.78
Initial SAPS2 (every 1 score increase)		1.02 (1.004–1.035)	0.01
AKI (vs. non-AKI)			0.002
	Stage 1	1.461 (0.770–2.772)	0.25
	Stage 2	1.497 (0.627–3.575)	0.36
	Stage 3	2.690 (1.472–4.915)	0.001

All above variables were inputted in multivariate Cox-proportional hazard regression analysis. ECMO, extracorporeal membrane oxygenation; ARDS, acute respiratory distress syndrome; VA, venoarterial; VV, venovenous; IABP, intraarterial balloon pump; RDW, red cell distribution width; SAPS2, Simplified acute physiology score 2; AKI, acute kidney injury.

**Table 3:** Calibration and discrimination analysis for in-hospital mortality and stage 3 AKI.

	Calibration			Discrimination					
	Hosmer-Lemeshow chi	df	*p*	AUROC ± SE	95% CI	*p*	Cut-off value	Sensitivity	Specificity
**For in-hospital mortality**									
SAPS2 (score)	11.520	8	0.174	0.612 ± 0.032	0.550–0.674	0.001	69.5	0.37	0.84
Pump speed (x10^3^ rpm)	11.691	8	0.166	0.597 ± 0.032	0.534–0.660	0.003	2.19	0.55	0.67
Sodium (mmol/L)	6.916	8	0.546	0.576 ± 0.032	0.512–0.639	0.021	147.6	0.28	0.89
**For stage 3 AKI**									
Pump speed (x10^3^ rpm)	15.492	8	0.050	0.569 ± 0.033	0.505–0.634	0.033	2.11	0.58	0.59
RDW (%)	10.519	8	0.230	0.668 ± 0.030	0.609–0.726	<0.001	14.1	0.71	0.53

SAPS2, Simplified acute physiology score 2; AKI, acute kidney injury; RDW, red cell distribution width; AUROC, area under the curve of receiver operating characteristics; SE, standard error

We compared clinical characteristics according to the mode of ECMO. The length of the hospital stay before ECMO insertion was shorter in patients with VA mode than in those with VV mode. The level of CRP was lower in the VA mode group than in the VV mode group. Nonetheless, SAPS2 was not different between the two groups. The initial ECMO settings were also comparable between the two groups. According to the linear regression analysis, there was no correlation between SAPS2 and ECMO speed either in VV mode (R^2^ = 0.003, p = 0.59) or VA mode (R^2^ = 0.001, p = 0.709). The mortality within 2 weeks after ECMO insertion was significantly higher in patients with VA mode than in those with VV mode (p = 0.03), whereas the overall in-hospital mortality was significantly lower in the VA mode group than that in the VV mode group (p = 0.02). Compared to the patients with the VV mode, those with the VA mode had shorter stays in the intensive care unit and hospital; however, there was no difference in the occurrence of AKI between the two groups (Table 4).

**Table 4:** Clinical characteristics according to the mode of ECMO.

		VV (n = 92)	VA (n = 230)	*p*
Length of stay before ECMO insertion (days)		17.3 ± 26.4	5.2 ± 11.7	<0.001
C-reactive protein (nmol/L)^a^		127.8 ± 92.0	46.9 ± 63.3	<0.001
SAPS2 (score) ^a^		61.1 ±15.0	61.1 ± 15.1	0.90
ECMO pump speed (10^3^ rpm) ^a^		2.3 ± 0.6	2.3 ± 0.6	0.88
ECMO blood flow rate (L/min)		3.0 ± 0.8	3.1 ± 1.0	0.51
Mortality within 2 weeks		22/92 (23.9)	84/230 (36.5)	0.03
In-hospital mortality		57/92 (62.0)	109/230 (47.4)	0.02
Intensive care unit stay (days)		35.9 ± 35.3	16.6 ± 42.2	<0.001
In-hospital stay (days)		61.7 ± 57.3	33.7± 51.2	<0.001
AKI		78/92 (84.8)	187/230 (81.3)	0.46
	Non- AKI	14/92 (15.2)	43/230 (18.7)	0.46
	Stage 1 AKI	26/92 (28.3)	79/230 (34.3)	0.29
	Stage 2 AKI	9/92 (9.8)	14/230 (6.1)	0.25
	Stage 3 AKI	43/92 (46.7)	94/230 (40.9)	0.34

Values are expressed as mean ± standard deviation in continuous variables and n/total (%) in categorical variables. Difference was analyzed by t-test in continuous variables and chi-square test in categorical variables. VV venovenous; VA, venoarterial; ECMO, extracorporeal membrane oxygenation; SAPS2, Simplified acute physiology score 2; AKI, Acute kidney injury.

^a^ The total numbers of VA/VV modes of C-reactive protein, SAPS2 and ECMO pump speed were 214/76, 221/91 and 229/92, respectively.

Because AKI, especially stage 3 AKI, showed a significant association with in-hospital mortality, we attempted to detect the risk factors associated with AKI and stage 3 AKI. We compared the characteristics between the patients with and without AKI. The initial ECMO pump speed was lower in those without AKI than in those with AKI. Those without AKI received ECMO support for a shorter period of time than those with AKI. The length of stay before ECMO insertion, BUN, total bilirubin, RDW and SAPS2 were associated with the occurrence of AKI. In the multivariate logistic regression analysis, the initial ECMO pump speed and the duration of ECMO support showed a statistical significance with ORs (95% CI, p-value) of 2.018 (1.129–3.609, p = 0.02) per 1,000 rpm in ECMO pump speed and 1.124 (1.035–1.22, p = 0.005) per day in duration of ECMO support (Table 5). These variables were also significant risk factors for developing stage 3 AKI (Table 6).

**Table 5:** Odds ratios for AKI.

		Univariate			Multivariate	
		Non-AKI (n = 57)	AKI (n = 265)	*p*	OR (95% CI)	*p*
Age (years)		58.4 ± 14.5	60.7 ± 15.5	0.29	1.013 (0.99–1.036)	0.28
Male sex (vs. female)		38/57 (66.7)	175/265 (66)	0.93	0.996 (0.503–1.857)	0.92
Center 2		27/57 (47.4)	147/265 (55.5)	0.27	-	-
Cause of admission				0.50	-	-
	CV disease	39/57 (68.4)	164/265 (61.9)	0.35	-	-
	Lung disease	9/57 (15.8)	40/265 (15.1)	0.90	-	-
	Malignancy	6/57 (10.5)	29/265 (10.9)	0.93	-	-
	Others	3/57 (5.3)	32/265 (12.1)	0.13	-	-
ECMO VA mode		43/57 (75.4)	187/265 (70.6)	0.46	-	-
IABP use		19/57 (33.3)	87/265 (32.8)	0.94	-	-
LOS before ECMO insertion (days)		4.3 ± 6.7	9.6 ± 19.5	<0.001	1.016 (0.982–1.052)	0.36
Initial ECMO Setting						
	Pump speed (10^3^ rpm) ^a^	2.1 ± 0.5	2.3 ± 0.7	0.01	2.018 (1.129–3.609)	0.02
	Blood flow rate (L/min)	2.9 ± 0.9	3.1 ± 0.9	0.09	-	-
	Blood flow rate in VA (L/min)	3.0 ± 1.0	3.1 ± 1.0	0.58		
	Blood flow rate in VV (L/min)	2.5 ± 0.8	3.1 ± 0.8	0.004		
ECMO duration (days)		5.0 ± 5.1	9.5 ± 10.1	<0.001	1.124 (1.035–1.22)	0.005
Initial urine output (L/day)		5.1 ± 4.9	4.1 ± 2.9	0.14	-	-
Initial ventilator settings						
	PEEP (cmH_2_O) ^a^	5.6 ± 3.0	5.9 ± 2.4	0.41	-	-
	PIP (cmH2O) ^a^	16.6 ± 6.1	17.4 ± 5.9	0.37	-	-
Initial laboratory findings						
	BUN (mmol/L)	14.9 ± 6.3	19.5 ± 12.4	<0.001	1.03 (0.987–1.074)	0.18
	Creatinine (μmol/L)	106.2 ± 48.1	113.3 ± 51.7	0.34	-	-
	Total bilirubin (μmol/L) ^a^	23.0 ± 19.9	31.8 ± 31.3	0.008	1.011 (0.995–1.027)	0.20
	Albumin (g/L) ^a^	28.5 ± 5.9	27.1 ± 6.4	0.15	-	-
	WBC (10^3^/ μL)	13.8 ± 6.4	13.8 ± 7.9	1.00	-	-
	Hemoglobin (g/dL)	11.3 ± 2.1	10.9 ± 2.4	0.23	-	-
	Platelet (10^3^/ μL)	140.4 ± 72.3	145.3 ± 86.2	0.69	-	-
	RDW (%)	14.2 ± 1.1	14.6 ± 1.8	0.02	1.001 (0.812–1.235)	0.99
	Sodium (mmol/L)	141.1 ± 6.7	141.5 ± 7.3	0.73	-	-
	Potassium (mmol/L)	3.9 ± 0.7	3.9 ± 0.8	1.00	-	-
	Chloride (mmol/L)	106.8 ± 8.0	105.8 ± 7.2	0.37	-	-
	CRP (nmol/L) ^a^	70.2 ± 80.1	67.6 ± 80.3	0.83	-	-
Initial SAPS2 (score) ^a^		57.1 ± 16.6	62.0 ± 14.5	0.03	1.014 (0.992–1.037)	0.21

Values are expressed as mean ± standard deviation in continuous variables and n/total (%) in categorical variables. Difference in univariate analysis was calculated by t-test in continuous variables and chi-square test in categorical variables. The reference of the continuous variables in multivariate analysis was every 1 unit increase of each variable. AKI, acute kidney injury; CV, cardiovascular; ECMO, extracorporeal membrane oxygenation; VA, venoarterial; VV, venovenous; IABP, intraarterial balloon pump; LOS, length of stay; PEEP, positive end expiratory pressure; PIP, peak inspiratory pressure; BUN, blood urea nitrogen; WBC, white blood cells; RDW, red cell distribution width; CRP, C-reactive protein; SAPS2, Simplified acute physiology score 2.

^a^The total numbers of AKI/non-AKI group of pump speed, PEEP, PIP, total bilirubin, albumin, CRP and initial SAPS2 were 264/57, 254/53, 253/53, 262/57, 263/57, 237/53 and 256/56, respectively.

**Table 6:** Odds ratios for stage 3 AKI.

		Univariate			Multivariate	
		Non-stage 3 AKI (n = 185)	Stage 3 AKI (n = 137)	*p*	OR (95% CI)	*p*
Age (years)		60.0 ± 14.5	60.7 ± 16.4	0.69	1.007 (0.99–1.025)	0.42
Male sex (vs. female)		123/185 (66.5)	90/137 (65.7)	0.88	0.985 (0.585–1.657)	0.95
Center 2		95/185 (51.4)	79/137 (57.7)	0.26	-	-
Causes of admission				0.35	-	-
	CV disease	119/185 (64.3)	84/137 (61.3)	0.58	-	-
	Lung disease	23/185 (12.4)	26/137 (19)	0.11	-	-
	Malignancy	20/185 (10.8)	15/137 (10.9)	0.97	-	-
	Others	23/185 (12.4)	12/137 (8.8)	0.30	-	-
ECMO VA mode		136/185 (73.5)	94/137 (68.6)	0.34	-	-
IABP use		70/185 (37.8)	36/137 (26.3)	0.03	0.983 (0.559–1.729)	0.95
LOS before ECMO insertion (days)		6.5 ± 12.9	11.6 ± 22.9	0.02	1.003 (0.988–1.018)	0.72
Initial ECMO Setting						
	Pump speed (10^3^ rpm) ^a^	2.2 ± 0.6	2.3 ± 0.7	0.03	1.576 (1.058–2.348)	0.03
	Blood flow rate (L/min)	3.0 ± 0.9	3.2 ± 1.0	0.14	-	-
	Blood flow rate in VA (L/min)	3.1 ± 0.9	3.2 ± 1.1	0.39		
	Blood flow rate in VV (L/min)	2.9 ± 0.8	3.2 ± 0.8	0.11		
ECMO duration (days)		6.4 ± 7.0	11.7 ± 11.5	<0.001	1.058 (1.019–1.098)	0.003
Initial urine output (L/day)		4.6 ± 3.7	3.9 ± 2.8	0.08	-	-
Initial ventilator settings						
	PEEP (cmH_2_O) ^a^	5.5 ± 2.4	6.2 ± 2.6	0.04	1.018 (0.914–1.134)	0.75
	PIP (cmH2O) ^a^	17.0 ± 6.1	17.5 ± 5.7	0.47	-	-
Initial laboratory findings						
	BUN (mmol/L)	17.5 ± 10.9	20.3 ± 12.5	0.04	1.006 (0.983–1.029)	0.60
	Creatinine (μmol/L)	108.5 ± 48.6	116.8 ± 54.2	0.15	-	-
	Total bilirubin (μmol/L) ^a^	26.9 ± 27.1	34.9 ± 32.6	0.02	1.008 (0.999–1.018)	0.08
	Albumin (g/L) ^a^	28.0 ± 6.5	26.6 ± 6.1	0.047	0.97 (0.932–1.01)	0.14
	WBC (10^3^/ μL)	13.9 ± 7.0	13.6 ± 8.5	0.71	-	-
	Hemoglobin (g/dL)	11.1 ± 2.4	10.7 ± 2.2	0.14	-	-
	Platelet (10^3^/ μL)	147.8 ± 81.9	139.8 ± 86.5	0.40	-	-
	RDW (%)	14.1 ± 1.2	15.1 ± 2.1	<0.001	1.308 (1.053–1.625)	0.02
	Sodium (mmol/L)	140.9 ± 7.0	142.1 ± 7.3	0.15	-	-
	Potassium (mmol/L)	3.9 ± 0.7	3.9 ± 0.8	0.54	-	-
	Chloride (mmol/L)	106.0 ± 7.5	105.9 ± 7.0	0.91	-	-
	CRP (nmol/L) ^a^	63.3 ± 74.5	74.6 ± 87.0	0.23	-	-
Initial SAPS2 (score) ^a^		60.4 ± 15.7	62 ± 14.1	0.35	-	-

Values are expressed as mean ± standard deviation in continuous variables and n/total (%) in categorical variables. Difference in univariate analysis was calculated by t-test in continuous variables and chi-square test in categorical variables. The reference of the continuous variables in multivariate analysis was every 1 unit increase of each variable. AKI, acute kidney injury; CV, cardiovascular; ECMO, extracorporeal membrane oxygenation; VA, venoarterial; VV, venovenous; IABP, intraarterial balloon pump; LOS, length of stay; PEEP, positive end expiratory pressure; PIP, peak inspiratory pressure; BUN, blood urea nitrogen; WBC, white blood cells; RDW, red cell distribution width; CRP, C-reactive protein; SAPS2, Simplified acute physiology score 2.

^a^The total numbers of stage3 AKI/non-stage3 AKI group of pump speed, PEEP, PIP, total bilirubin, albumin, CRP and initial SAPS2 were 137/184, 132/175, 132/174, 134/185, 135/185, 124/166 and 130/182, respectively.

There was an additional risk factor in stage 3 AKI. The RDW was significantly lower in those without stage 3 AKI than in those with stage 3 AKI. In the multivariate logistic regression analysis, the RDW was still statistically significant, with an OR (95% CI, p-value) of 1.308 (1.053–1.625, 0.02) for every 1% increase (Table 6). In the calibration and discrimination analysis, stage 3 AKI was well-calibrated and discriminated by a cut-off value of 14.1% for RDW (Table 3). We compared patient characteristics according to the RDW status. Patients with an RDW above 14.1% showed significantly higher level of CRP than did those with an RDW below 14.1%. Moreover, patients with an RDW above 14.1% showed considerably lower hemoglobin, mean corpuscular volume, mean corpuscular hemoglobin, and mean corpuscular hemoglobin concentration than did those with an RDW below 14.1% (Table 7).

**Table 7:** Patient characteristics according to the status of RDW.

	RDW < 14.1% (n = 138)	RDW ≥ 14.1% (n = 184)	*p*
Age (years)	60.5 ± 15.3	60.2 ± 15.4	0.83
Male sex	96/138 (69.6)	117/184 (63.6)	0.26
Length of stay before ECMO insertion (days)	4.8 ± 11.7	11.5 ± 21.2	<0.001
ECMO VA mode	110/138 (79.7)	120/184 (65.2)	0.004
C-reactive protein (nmol/L)^a^	51.9 ± 72.1	80 ± 83.7	0.002
Blood urea nitrogen (mmol/L)	17.2 ± 11.2	19.8 ± 11.9	0.04
Total bilirubin (μmol/L)	22.7 ± 21.6	36 ± 33.6	<0.001
Hemoglobin (g/dL)	11.6 ± 2.3	10.5 ± 2.2	<0.001
Mean corpuscular volume (fL)	91.7 ± 4.7	90.3 ± 6.3	0.03
Mean corpuscular hemoglobin (pg/cell)	30.9 ± 1.4	30 ± 2.3	<0.001
Mean corpuscular hemoglobin concentration (g/dL)	33.8 ± 1.2	33.2 ± 1.3	<0.001
RDW (%)	13.4 ± 0.5	15.4 ± 1.8	<0.001
ECMO duration (days)	6.3 ± 5.7	10.4 ± 11.3	<0.001
Intensive care unit stay (days)	14.7 ± 16.2	27.7 ± 52.1	0.002
In-hospital stay (days)	31.2 ± 27.5	49.5 ± 67	0.001

Values are expressed as mean ± standard deviation in continuous variables and n/total (%) in categorical variables. Difference was analyzed by t-test in continuous variables and chi-square test in categorical variables. ECMO, extracorporeal membrane oxygenation; RDW, red cell distribution width; VA, venoarterial.

^a^ Total numbers of RDW ≥/< 14.1% groups of C-reactive protein and total bilirubin were 167/123 and 181/138, respectively.

## Discussion

In this work, we investigated the risk factors of AKI and in-hospital mortality in patients receiving ECMO support. Here, we found that the initial pump speed of ECMO was associated with in-hospital mortality and AKI. The elevated RDW could be suggested as the risk factor for severe AKI in these patients. This was the first study to identify the risk factors of AKI in adult patients receiving ECMO support. Because AKI is the most common complication and a major risk factor of mortality, defining the risk factors for AKI in these patients is extremely important [9–14]. This study is the largest ECMO assessment ever reported. Moreover, the association of pump speed with AKI and mortality is a novel finding.

We showed that AKI, especially stage 3 AKI, was a significant risk factor for in-hospital mortality in patients receiving ECMO support. SAPS2 and serum sodium level were also important risk factors of in-hospital mortality. Along with these well-known and expected findings [11–13, 18–20], we found that the initial pump speed of ECMO was significantly related to in-hospital mortality, with a 33% increased risk for every 1,000 rpm increase. The initial pump speed of ECMO was also a risk factor for both AKI and stage 3 AKI. On the other hand, the blood flow rate of ECMO was not associated with in-hospital mortality or AKI. Why a high pump speed, but not a high blood flow rate of ECMO, increases the risk of in-hospital mortality and AKI is not clear at this time. However, the ECMO pump can induce hemolysis, leukocyte and platelet destruction, and complement activation [21, 22]. Blood flow through the ECMO circuit is driven by centrifugal pump. A rotating impeller in centrifugal pumps spins, which creates a constrained vortex that suctions blood into the pump and propels it out toward the membrane oxygenator [23]. Hemolysis has been reported to be associated with AKI [24]. In addition, Lou et al. found that the pump speed was a risk factor for hemolysis and that hemolysis was associated with adverse outcomes in pediatric patients receiving ECMO [25]. Although we did not evaluate the degree of hemolysis in our patients, we postulate that hemolysis caused by high revolutions of the ECMO pump might result in AKI and in-hospital mortality. To provide stable cardiac output in the VA mode and adequate oxygenation in the VV mode, adequate blood flow should be maintained. Therefore, clinicians raise the ECMO pump speed as much as possible to maintain adequate blood flow. The blood flow rate that was applied to 90% of our patients was less than 4.1 L/min. A high blood flow extracorporeal circuit that pumped up to 7 L/min [26] did not apply to our patients; however, 43.8% (141/321) of our patients were treated with a pump speed higher than the cut-off value of 2.19 x 10^3^ rpm. For these reasons, we speculate that pump speed, but not a blood flow, is a predictor of death in this study.

We compared the clinical characteristics of patients from the VA and VV ECMO modes. Patients with the VV mode had higher levels of CRP, showed higher mortality, and had longer stays in the hospital compared with those with the VA mode; however, the mortality within 2 weeks after ECMO insertion was higher in patients with the VA mode. We speculated that the patients with the VA mode deteriorated rapidly but recovered soon if they were not severe enough for death. In contrast, patients with the VV mode seemed to show slower but poorer outcomes than those with the VA mode. The different disease process of the patients treated with the VA and VV ECMO modes [27] might be related to these findings. Future prospective studies will be needed to investigate whether ECMO mode determines outcomes.

In this study, the higher the RDW was, the more frequently stage 3 AKI occurred. To the best of our knowledge, this is the first study to suggest a potential role of the RDW in AKI. Recently, the use of the RDW as a simple and inexpensive biomarker to predict mortality in chronic heart failure [18, 28], liver disease [29], and critical illness [30] has increased. Moreover, the RDW has been reported to be associated with many pathological conditions such as colon cancer, inflammatory bowel disease, celiac disease, rheumatoid arthritis, Alzheimer’s disease, and contrast-induced nephropathy [31, 32]. Although the exact mechanism of this relationship is not clear, inflammation is a proposed underlying factor [33, 34]. This proposed factor can also be supported by our data, which indicate that the elevated RDW was associated with high CRP levels in the patients. In this study, patients with an RDW greater than 14.1% showed lower RBC indices than did those with an RDW less than 14.1%. Because anemia is a risk factor for AKI [35], the low RBC indices found in the elevated RDW group might contribute to increase the odds of stage 3 AKI occurring.

The current study suffered from several limitations. First, this study is a retrospective cohort study; however, the variables before ECMO insertion were well retrieved with a less than 10% missing rate. Moreover, this is the largest study to explore the association of AKI and mortality in patients receiving ECMO support [11–13]. A low level of missing data and a large number of patients could partially compensate for the weakness of the study design. Second, we classified the patients into their KDIGO stage based only on their serum creatinine concentration. Urine volume is a sensitive marker for the early detection of AKI in patients on ECMO. Decreased urine volume during ECMO treatment and/or on the day of ECMO removal can be attributed to decreased cardiac output following decannulation, and can be correlated with acute cardiorenal syndrome type 1 [27, 36, 37]. Third, we could not provide direct evidence that hemolysis due to a high pump speed resulted in AKI in this study. We should have measured plasma-free hemoglobin, which is an indicator of hemolysis. Furthermore, we did not obtain information on the cannulation site and mean venous pressure in the ECMO circuit. Finally, this study was composed of data from two centers, which could limit the generalizability.

In conclusion, AKI is a significant risk factor for in-hospital mortality in patients receiving ECMO support. The initial pump speed of ECMO is associated with in-hospital mortality and strongly related to AKI, especially stage 3 AKI. Therefore, once adequate blood flow is maintained, clinicians must be careful not to further increase the ECMO pump speed. Because the elevated RDW was also strongly related to stage 3 AKI, special attention should be paid to patients with abnormal RDW values to prevent AKI.
